# Knowledge is Power: Challenging Ageist Attitudes as a Pathway to Careers in Aging

**DOI:** 10.1093/geroni/igaf122.2206

**Published:** 2025-12-31

**Authors:** Hannah Friedland, Jillian Crocker, Amanda Morgan, Jessica Ruiz, Soledad Arguelles-Borge, Ashley Stripling

**Affiliations:** Nova Southeastern University, Fort Lauderdale, Florida, United States; Nova Southeastern University, Fort Lauderdale, Florida, United States; Nova Southeastern University, Fort Lauderdale, Florida, United States; Nova Southeastern University, Fort Lauderdale, Florida, United States; Nova Southeastern University, Fort Lauderdale, Florida, United States; Nova Southeastern University, Fort Lauderdale, Florida, United States

## Abstract

Ageist bias is underrecognized and contributes to misdiagnosis, delayed treatment, and a range of adverse mental health outcomes for older adults. Aging-related coursework and awareness of bias may lead to reductions in ageist attitudes. Therefore, the current study aims to examine the impact of implicit bias assignments and subsequent knowledge from an eight-week adult and older adult development course taught by a geropsychologist. Participants consisted of 26 first-year psychology doctoral students asked to complete pre/post measures of the Age Harvard Implicit Association Test (IAT) and a written reflection. Results of the IAT were coded on a 7-point Likert scale to assess the prevalence and change of preferences for young people or older people. The reflections were analyzed using constructivist grounded theory methodology. Descriptive statistics revealed that 77.8% of participants had a baseline preference for young people. Notably, a paired-samples t-test indicated that pre-test scores (M = 2.58, SD = 1.137) were significantly different than post-test scores (M = 3.38, SD = 1.134), t(25)=-3.035, p=.003, suggesting a shift in scores towards age neutrality. On the baseline reflection, participants reported a range of emotional responses (e.g., shock, disbelief), rationalizations (e.g., fear of aging, lack of exposure to older adults, sociocultural influences), and acknowledged the impact of bias on professional practice. At post-test, participants reported a shifted perspective on aging due to course content, with some indicating novel career interest in geropsychology. Implications of the current presentation emphasize the importance of implementing aging-related coursework and awareness of bias early in graduate training to develop geriatric interest and competency of care.

